# Evaluating the integration of chronic care elements in primary health care for people with mental illness: a longitudinal study in Nepal conducted among primary health care workers

**DOI:** 10.1186/s12913-020-05491-0

**Published:** 2020-07-09

**Authors:** Nawaraj Upadhaya, Mark J. D. Jordans, Ramesh P. Adhikari, Dristy Gurung, Ruwayda Petrus, Inge Petersen, Ivan H. Komproe

**Affiliations:** 1 Transcultural Psychosocial Organization Nepal, Kathmandu, Nepal; 2 Department of Research and Development, War Child, Amsterdam, the Netherlands; 3 Centre for Global Mental Health, Institute of Psychiatry, Psychology and Neuroscience, King’s College London, London, UK; 4 School of Applied Human Sciences, University of KwaZulu-Natal, Durban, South Africa; 5 Centre for Rural Health, College of Health Sciences, University of KwaZulu-Natal, Durban, South Africa; 6 Department of Research and Development, HealthNet TPO, Amsterdam, the Netherlands; 7 Utrecht University, Utrecht, the Netherlands

**Keywords:** Chronic care, Mental health, Nepal, Primary health care, Health workers

## Abstract

**Background:**

Despite many important developments in the global mental health arena in the past decade, many people with mental health problems still do not have access to good quality mental health care. The aim of this study was to evaluate the perceived impact of a mental health care package (MHCP) in integrating chronic care elements in primary health care for people with mental illness.

**Methods:**

A controlled pre-post study design was used in 20 primary health care facilities in Chitwan, Nepal. We compared 10 health facilities that had implemented a MHCP (intervention group), with 10 health facilities that had not implemented the MHCP (comparative control group) but provided regular physical health services. We administered the Assessment of Chronic Illness Care (ACIC) tool on a group basis within all 20 health facilities among 37 health workers. Data was collected at three time points; at baseline, midline (at 13 months from baseline) and end line (at 25 months from baseline).

**Results:**

From baseline to end line, we see a notable shift in the level of support reported by the intervention health facilities compared to those in the comparative control group. While at baseline 10% of the intervention health facilities had basic support for the implementation of chronic illness care, at the end line, 90% of the intervention group reported having reasonable support with the remaining 10% of the intervention facilities reporting that they had full support. In contrast, 20% of the health facilities in the comparative control group at end line still reported having limited support for the implementation of chronic illness care, with the remaining 80% only managing to shift to the next level which is basic support.

**Conclusions:**

These findings suggest that training and supervision of primary health care workers in the implementation of MHCP interventions can lead to strengthening of the system to better address the needs of patients with chronic mental health problems. However, substantial financial and coordination inputs are needed to implement the MHCP. The comparative control group also demonstrated improvements, possibly due to the administration of the ACIC tool and components of counselling services for family planning and HIV/AIDS services.

## Background

The past decade has seen many important contributions in the global mental health arena, including the Lancet series 2007 and 2011 on mental health, the Inter Agency Standing Committee guidelines for Mental Health and Psychosocial Support in emergency settings, and World Health Organization (WHO)'s Mental Health Gap Action Program (mhGAP) [1]. Despite this progress, globally the gap between the number of people who have a mental disorder and those who receive treatment for their disorder remains high and is commonly referred to as the ‘treatment gap’ [2]. In low resourced countries the treatment gap is fast increasing to nearly 90% [3]. The WHO’s World Mental Health Survey reported that between 76.3 to 85.4% of severe mental health cases in less-developed countries received no treatment 12 months preceding participation in the survey [4]. One explanation for this high treatment gap is the lack of trained human resources in mental health. A meta-analysis finds that all low income countries and 59% of middle income countries had far fewer mental health professionals than what was needed [5].

To help bridge the treatment gap, a task-sharing approach has been advocated by the WHO, where non-specialist health workers are trained to provide predetermined packages of mental health interventions and counseling under clinical supervision which is provided by mental health specialists [6]. This collaborative care approach involving generalists and specialists can also yield positive socio-economic and health outcomes for people with mental, neurological and substance abuse (MNS) disorders [7]. The task-sharing approach has been found to be acceptable and feasible when pre-conditions such as adequate training and compensation to primary health care workers, better access to psychotropic drugs and provision of structured supervision are in place [8].

Mental illness often requires chronic care. Therefore, the focus on perceived quality of care from the chronic care model (CCM) is important for health systems to be able to respond to the needs of patients with chronic mental illness and bridge the gap between the provisions of care and need for treatment. Collaborative care does, however, require coordination of care across providers. The collaborative CCM provides a framework for enabling such coordination as well as enabling the health system to respond to both the acute and chronic nature of MNS disorders. In high income countries, these collaborative CCMs in primary health care settings have resulted in positive patient outcomes for MNS disorders [9]. The CCM involves interventions to support patient’s self-management, help service providers to make decisions on treatment plans, link patients and family members to available resources in the community, support the design of systems for health service delivery, organizational structures and clinical information systems [10]. Furthermore, the CCM provides practical strategies for integrated mental health care [11]. The review of research evidence shows that redesigning care using the CCM model improves patient care and yields better health outcomes [12], and reduces health care costs [13]. The CCM has been found useful not only for chronic disease management in primary care but also for the prevention of risky health behaviors which causes chronic illness [14]. Yet, few LMICs have assessed the need for collaborative CCMs for mental health problems, or reviewed the ways in which integrated mental health care can produce a positive impact on supporting the development of collaborative chronic care.

In Nepal, to the best of our knowledge, a CCM has not yet been applied for the provision of mental health care. However, several initiatives taken by non-governmental organizations in mental health and psychosocial training, service delivery, clinical supervision and mental health and psychosocial research show that some components of CCM have inadvertently been applied [15]. One such initiative is the implementation of the mental health Gap Action Program (mhGAP) based mental health care package (MHCP) which focuses on a task sharing approach. As part of the Program for Improving Mental Health Care (PRIME) project, MHCP was developed and implemented in the Chitwan district by Transcultural Psychosocial Organization Nepal and Ministry of Health [7]. While this program did not fully implement the CCM, it contained several interventions that promoted the CCM elements such as decision support for service providers, mental health information system, community linkages, and patient self-care.

Study aims:

- To identify the application and gaps in the organization and implementation of chronic collaborative care for priority mental disorders (depression, alcohol use disorder and psychosis) at the primary health facility level.
- To assess whether the implementation of the MHCP was a feasible and effective strategy for integrating chronic care elements in primary health care for people with mental illness.

## Methods

### Study design

A controlled pre-post study design using provider self-report on the organization of the health care system according to the elements of CCM was used.

### Setting and sampling

The study sites (*n* = 20) were primary health care facilities in Chitwan, central Nepal.

In 10 health facilities of Western Chitwan the MHCP was being implemented and piloted hence these health facilities became the intervention group by default. For comparison, we needed another 10 health facilities having similar baseline characteristics but being geographically far from the intervention health facilities to reduce the chances of contamination. These 10 health facilities from Eastern Chitwan (i.e. comparative control group) were selected by district based program staff based on travel distance, accessibility, and population density. The control sites were the health facilities where MHCP was planned to be scaled up following the completion of all the three measurements (baseline, midline and end line) of the study.

The health facilities in the intervention group implemented the MHCP (described in Table 1) for depression, epilepsy, psychosis, and alcohol use disorders during the study period. The health facilities in the comparative control group did not receive any mental health care support during the study period, but provided regular physical health services (care as usual, which included general outpatient consultation for common physical diseases, antenatal and post natal care, delivery services, immunization services, family planning services and the care for HIV/AIDS and tuberculosis). The details of the MHCP has been published in detail elsewhere [7]. A summary of the activities involved in the implementation of the MHCP as they relate to the elements of the CCM, is presented in Table 1.

**Table 1:** Interventions under the mental health care package categorized as per the elements of chronic care model

Chronic care model elements	Interventions under the Mental Health Care Package (MHCP)
Organization of the Healthcare Delivery System	1) Involvement of clinicians, civil society organization and policy makers in the development of treatment protocol for delivery of mental health services; 2) Participatory process of development of mental health training guidelines and accreditation from National Health Training Center; 3) Policy engagement workshops at the national and district levels; and 4) Managerial supervision by District Public Health Office (DPHO).
Community linkages	1) Anti-stigma program, mass sensitization and awareness through radio program; 2) Home based care by FCHVs; 3) Psychosocial counseling by community based counselors; and 4) Use of Community Informant Detection Tool (CIDT) by community members to identify and refer people with mental health and psychosocial problems.
Self-care management support for patients	1) Psycho-education on self-care management strategies, stress and anger management techniques, common side effects of psychotropic drugs and consequences of inappropriate use of drugs; 2) Individual patient goal setting through counseling; 3) Relaxation exercises; and 4) Peer-support.
Decision support for service providers	1) Diagnosis and treatment plan flow chart; 2) Checklist for screening suicidal ideation, depression, epilepsy, psychosis and alcohol use; 3) Monthly clinical supervision to discuss on difficult cases; and 4) Provision of phone contacts to psychiatrist and clinical psychologist for consultation of difficult cases.
Delivery system redesign	1) Appointment system for follow up; 2) Defaulters tracking system; 3) Psychotropic drug supply chain system; and 4) System for supervision/onsite coaching and referral pathways.
Clinical information system	1) Out Patient Department (OPD) card and patient register; 2) Monthly data compilation using data from patient registers; 3) Drug quantification, storage, recording and drug demand and supply tracking system; and 4) Integration of mental health indicators into existing health management information system.

Prescribers (primary health care workers such as medical officers, health assistants and community medical assistants) received 9 days of training on basic mental health care such as diagnosis, psycho-education, drug prescription and side effect management based on the mhGAP intervention guide [1]. Non-prescribers (such as nurses and auxiliary nurse midwives) received 10 days of training on basic psychosocial support and psychosocial intervention protocols such as the brief version of behaviour activation-based Healthy Activity Program for depression [16]; motivational interviewing-based Counseling for Alcohol Abuse disorders [17]. The community-based health workers known as Female Community Health Volunteers (FCHVs) were trained for 2 days on identification and referral of mental health cases using a Community Informant Detection Tool, which is a structured tool for proactive case detection [18, 19]. The FCHVs were also trained on how to conduct community level mass sensitization and anti-stigma programs and how to support families to ensure treatment adherence through home-based care. The MHCP also included psychological treatment, mainly counseling support (healthy activity program for depression and counseling for alcohol problems) provided by community counselors who visited the clients (patients) in the community and provided psychosocial support. Besides the initial classroom based trainings, regular clinical supervision (of prescribers by the psychiatrist, of non-prescribers and community psychosocial counselors by clinical psychologist and of FCHVs by community psychosocial counselor), case conferences (an opportunity to observe a senior psychiatrist/psychologist seeing the client in front of the prescribers) and provision of on-site coaching during field visits were implemented to strengthen the MHCP.

The policy engagement workshops and group meetings were organized to orient policy makers and planners about mental health in general and the challenges of developing a system of care for mental illness, in particular. The health facility staff (both prescribers and non-prescribers) were trained on how to do mental health record keeping. A separate mental health register was developed and health workers were trained on how to complete those registers.

### Instrument and data collection procedures

The Assessment of Chronic Illness Care (ACIC), a quality improvement tool [10], is generally used to evaluate the strengths and weaknesses of delivery of care for chronic illness. In this study the ACIC was used to assess whether the MHCP was beneficial in integrating chronic care elements in primary health care for people with mental illness. The translation of the ACIC instrument from English to Nepali went through several steps of translation and cultural adaptation by a group of bi-lingual researchers. The ACIC is a service provider (health worker) self-assessment tool which consists of 31 items covering the six domains of the CCM, and a separate component to determine the level of integration of the CCM. Each domain of ACIC is independent from each other and provides specific health system information. The elements/components of the ACIC include: health care delivery system (4 items); community linkages (4 items); self-management support for patients (4 items); decision support for service providers (4 items); delivery system redesign (6 items); clinical information system (4 items), and integration of CCM elements (5 items). The health care delivery system component assesses the perception at the overall health systems components that play vital roles in supporting the system of care for chronic mental illness. Delivery system redesign component assesses the perception at how roles and responsibilities of team members can be reorganized so that they can work at an optimal level. The community linkages component refers to the coordination with community structures in order to help patients and family members access community resources. The self-management component assesses how patients and carers can be supported to adopt healthy behaviors and be acquainted with locally available self-care strategies. The decision support component covers the mechanisms to optimize service providers’ knowledge and skills to provide best possible services to people with MNS disorder. The clinical information systems component assesses the perception at how high quality data can be collected and analyzed to facilitate optimal clinical care and follow up. Finally, the integration of chronic care elements considers how such elements can be integrated into routine health service delivery.

Responses to items on the ACIC requires a score between 0 and 11. Scores of 0–2 are categorized as ‘little support for chronic illness care’, 3–5 indicate ‘basic support for chronic illness care’, 6–8 are categorized as ‘reasonably good support for chronic illness care’ and 9–11 indicate fully developed chronic illness care.

One ACIC form was administered per health facility by a team of experienced researchers with university education and training in mental health research, including the use of the ACIC questionnaire. Two health workers (one with clinical experience and another with administrative experience) per health facility provided one response (i.e. group rating) after discussing among themselves and arriving at a consensus rating for each item of the ACIC. Group response is preferred in ACIC because of its advantage of group reflection and representation of a true picture of the health facility [10]. As the care is not provided in isolation but in collaboration, the group rating was used to incorporate everyone’s views in assessing the system of care for mental illness in their health facilities and to evaluate whether MHCP improved the system of care for chronic mental illness. The number of health workers in each interview varied depending on availability but, on an average, two health workers provided collective answers per health facility. The types of health workers involved in answering the questions were: Primary Health Care Doctor (medical officer), Health Assistants, Nurse, Auxiliary Nurse Midwives, and Community Medical Assistants. The ACIC was administered three times (baseline in March 2014, followed by midline in April 2015, and end line in April/May 2016).

### Data analysis

The ACIC data was collected on paper forms and entered into SPSS. To verify the consistency of the data entered, we performed checks of all entered data by comparing the values with the paper version. To identify the missing values, the frequency table of each ACIC item was examined. The examination of frequency tables showed that there were no missing values. Descriptive statistics were used to calculate the mean scores and standard deviations of each sub-scale. The sub-scales mean for each domain of ACIC were calculated for the six sections of the ACIC and the overall program score (integration of care) for the baseline, midline and end line data. To determine longitudinal change of the ACIC domains, we performed univariate analysis of variance (ANOVA) with repeated measurements. We used Box’s M test to correct for deviations of normality and Mauchly’s W test to correct for sphericity.

## Results

The health facilities in intervention and comparative control groups had more or less similar baseline characteristics as presented in Table 2.

**Table 2:** Health facility characteristics at the baseline

Health Facility characteristics	Intervention Health Facilities	Comparative Control Health Facilities
Type of health facilities:	No. of sub-health post = 5 No. of health post = 4 No. of primary health care center = 1	No. of sub-health post = 3 No. of health post = 5 No. of primary health care center = 2
Health workers categories	No. of auxiliary health worker = 25 No. of auxiliary nurse midwives = 20 No. of staff nurse = 2 No. of health assistant = 4 No. of medical officer = 3	No. of auxiliary health worker = 23 No. of auxiliary nurse midwives = 22 No. of staff nurse = 5 No. of health assistant = 6 No. of medical officer = 5
Average number of patients seen (daily)	Minimum number of patients seen daily = 33 Maximum number of patients seen daily =78	Minimum number of patients seen daily = 35 Maximum number of patients seen daily =96
Types of health services available	Primary care consultations and medication for skin disease, minor wounds, headache, fever etc. Antenatal, delivery and post natal care; immunization,	Primary care consultations and medication for skin disease, minor wounds, headache, fever etc. Antenatal, delivery and post natal care; immunization,
Availability of specialized delivery services	Number of birthing centers =3	Number of birthing centers =4
Health facility infrastructure	No. of old buildings = 9 No. of new buildings = 1	No. of old buildings = 7 No. of new buildings = 3

At baseline, there was no visible difference in ACIC sub-scores between intervention and comparative control health facilities, and both groups fell below the threshold for basic support (score between 3 and 5). The highest sub-scale mean score at baseline for the intervention group was for patient support (3.0). For the comparative control group the highest mean score was for delivery system design (3.2).

The ACIC was repeated at 13 months (i.e. midline) to establish whether there was any change in the mean scores after the implementation of the MHCP intervention. From Table 3, it is evident that at midline the intervention group reported much higher mean scores in all six elements of the ACIC. The most notable change being for decision support for service provider component, which saw a change from 1.1 at baseline to 7.8 at midline. For the comparative control group, there was also an improvement in the mean score on all six ACIC elements, the delivery system design component having the highest mean score of 3.9 (compared to its mean score of 3.2 at the baseline). However, the difference in the mean score from baseline to midline for the comparative control group was minimal when compared to the intervention group.

**Table 3:** System level changes from baseline to end line

Components	Baseline mean (SD)		Midline mean (SD)		End line mean (SD)		ANOVA (df, error time) = F statistic, *p*-value
	Intervention	Control	Intervention	Control	Intervention	Control	
Part 1: Organization of the Healthcare Delivery System	2.4 (1.1)	1.8 (0.6)	6.3 (2.1)	2.5 (1.6)	7.9 (1.1)	3.7 (1.6)	F(2,36) = 10.07, *p* < 0.001
Part 2: Community Linkages	2.3 (1.6)	1.4 (0.7)	7.1 (1.6)	2.7 (2.3)	8.5 (1.0)	3.4 (2.1)	F(2,36) = 11.31, *p* < 0.001
Part 3: Practice Level							
3a: Patient Support	3.0 (1.0)	2.6 (0.6)	7.0 (2.0)	3.9 (1.6)	8.1 (1.4)	3.9 (1.1)	F(2,36) = 11.58, *p* < 0.001
3b: Decision Support for Service Providers	1.1 (1.1)	0.6 (0.5)	7.8 (2.0)	1.7 (1.7)	8.2 (0.7)	4.7 (2.4)	F(2,36) = 17.55, *p* < 0.001
3c: Delivery System Design	2.9 (1.1)	3.2 (1.2)	7.5 (1.4)	3.9 (2.0)	8.2 (1.3)	4.5 (1.3)	F(2,36) = 12.34, *p* < 0.001
3d: Clinical Information Systems	1.8 (1.6)	1.4 (0.9)	7.3 (2.3)	2.7 (1.9)	8.4 (1.2)	2.9 (1.4)	F(2,36) = 12.98, *p* < 0.001
Part 4: Integration of Chronic Care Model Components	0.3 (0.7)	0.3 (0.6)	6.5 (2.1)	2.1 (2.2)	7.1 (1.5)	2.2 (1.3)	F(2,36) = 16.36, *p* < 0.001

At end line, conducted at 25 months (after 2 years of MHCP implementation), the intervention group made further progress in all six elements of the ACIC. The most notable change being for the community linkages component which saw a change from 7.1 at midline to 8.5 at end line. The comparative control group also had some improvements in all six elements of the ACIC. The most notable change being for the decision support for service provider component which saw a change from 1.7 at midline to 4.7 at end line.

### Integration of chronic care model elements

**Fig. 1:**
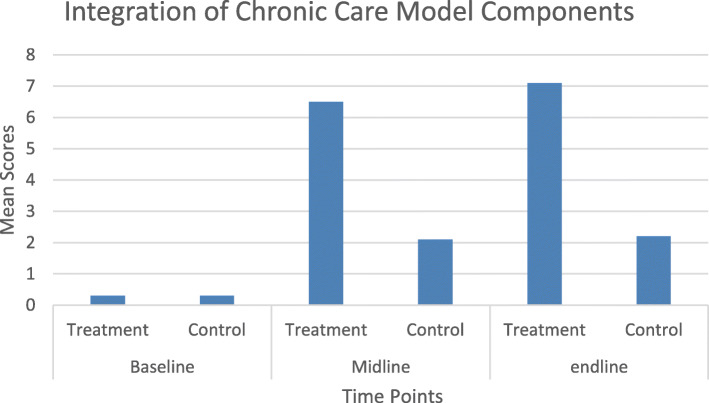
Integration of chronic care model components as a result of MHCP

The overall score of the ACIC (Fig. 1), which reports the extent of support for the integration of CCM elements, shows that the MHCP had positive and significant effects on strengthening elements of the CCM in primary health care facilities providing mental health services compared to the comparative control facilities.

At baseline both groups scored 0.3 for the integration of CCM elements, which is the lower limit threshold for little support (score between 0 and 2) of chronic care. These scores indicate that at baseline the health facilities in the intervention and comparative control groups had little support for the implementation of chronic illness care. At midline the intervention group showed remarkable improvements (the mean score changed from 0.3 at baseline to 6.5 at midline) with all health facilities moving from little support to reasonably good support for system of care for chronic mental illness. The health facilities in the comparative control group had some change of scores from baseline (0.3) to midline (2.1) but it was minimal compared to the intervention group; the comparative control group only moved from little support to basic support. At end line, the intervention group showed further improvements in mean score from midline (6.5) to end line (7.1), whereas for the health facilities in the comparative control group, the change was minimal from midline (2.1) to end line (2.2).

When these improvements were categorized as per the ACIC categories (little support, basic support, reasonably good support and full support), using mean of the sub-scale means, it showed that at baseline 90% of the health facilities in the intervention group, and 100% of the health facilities in the comparative control group had limited support for chronic illness care. At end line, 90% of the health facilities in the intervention group showed reasonably good support, and 10% had a fully developed chronic illness care system for mental illness. In the comparative control group, 20% of the health facilities remained in the same category (having limited support to chronic care illness) as in baseline, while 80% of them moved to the next category (having basic support for chronic illness care).

Table 3 presents the comparison of scores between intervention and comparative control group and demonstrates that a statistically significant effect was obtained for all sub-sections of the ACIC (*P* < 0.001), indicating that the health facilities in the intervention group had made statistically significant improvements in the elements of chronic care service delivery as they pertain to mental illness compared to the comparative control group.

## Discussion

The findings show that the MHCP was perceived to have had a positive impact on quality of care systems for chronic mental illness, through the integration of elements of the CCM into routine primary health care. At baseline, health facilities in both groups typically had little support for chronic mental illness care, but after implementation of MHCP, the facilities in the intervention group showed significant improvements on all domains of the ACIC, compared to health facilities in the comparative control group. For example, the higher mean score changes in intervention health facilities shows that essential elements for effective chronic care (such as clinical information system, patient support, decision support for service provider, and integration of care) were strengthened as a result of interventions under the MHCP.

The primary health workers’ acknowledgement of greater support for information system showed that training primary health care workers and the availability of mental health registers and proforma indicators improved the patient information system. If the current achievements in the clinical information systems can be sustained, the system will be able to provide quality information that is relevant for policy and practice especially in situations where countries in LMICs do not have reliable mental health information [20].

Another chronic care element that showed a large improvement in health workers’ assessment was patient self-care. The higher mean score change in support to patient self-care showed that the implementation of the MHCP brought about changes in the system of care in relation to how patients were supported at the health facility and linked to the community resources. For this to be sustained, linkages and coordination mechanisms with relevant stakeholders developed through the implementation of the MHCP need to be continued. Cramm and colleagues (2012) in the Netherlands showed that the delivery of chronic care improved due to improvements in coordination among professionals from diverse disciplines [21]. Coordination and collaboration between the health facility and community is vital to address supply and demand issues of mental health care [7]. The findings show that through the MHCP, the system was developed to support the patient self-care component of the CCM. Emphasis on self-care strategies during training and supervision sessions might have motivated health workers to provide information about self-care during interaction with patients. Additionally, regular home visits by FCHVs might have encouraged patients and caregivers to practice better self-care strategies, which are important pillars of a system of care for mental illness.

The higher mean score change for decision support reflects that through the MHCP the system was better organized, and primary health care workers were sufficiently trained and supervised to provide integrated mental health care at the primary health care level based on the CCM model. The availability of mhGAP guidelines in the Nepali language, regular training, supervision and provision of a monthly case conference with senior psychiatrists and psychologists might have helped primary health care workers to make better decisions in terms of diagnosis, treatment plan, and follow up of patients with mental illness. Sustained improvement, however, will depend upon whether decision support is adopted as part of the integrated support system. The assessment of chronic care for diabetic patients in primary health care settings in South Texas (USA) also found that other aspects of chronic care management needed to be present for the decision support for service provider elements of CCM to be effective [22].

The changes in mean scores for the integration of CCM elements show that at the primary health care level it is possible to develop organizational support structures to assist primary health care workers to provide more holistic integrated chronic care. However, this finding should be interpreted with caution as in this study a fully-fledged implementation of the CCM did not take place, and the assessment only provided the perspectives of primary health care workers on the system of care for chronic mental illness. Nevertheless, the findings show that primary health care workers were ready and willing to integrate the essential elements of chronic care within existing primary health care. This positive attitude of the primary health care workers opens avenues for the integration of mental health into primary health care in Nepal, including the elements of the CCM [1].

The study also found a trend for positive changes in the comparative control group. The improvements, though small, in health facilities in the comparative control group may be because of some of the CCM components in physical health programs and the testing effect of ACIC interview process. Although the health facilities in the comparative control group did not receive support as per the MHCP, the usual primary health care for physical health problems was being provided (in both intervention and comparative control health facilities), which had components of counseling, patient information system, inter-sectoral collaboration, and community linkages. The family planning, tuberculosis and HIV/AIDS programs in particular had components of counseling, so the health workers in the control health facilities might have received psychosocial knowledge and skills by participating in the trainings for those programs. The health workers might have applied this knowledge and skills because of the testing effect of the ACIC interview process, which allowed them to reflect on the situation of chronic illness care, identify the areas of improvement and develop collegial relationship by coming up with a group response to ACIC sub-scale items.

Previous studies have found that participatory reflective practice and collegial environments send clear messages to staff about the importance of chronic illness care and thereby, contribute to the improvements in health care practices and patient outcomes [23]. The awareness created by the ACIC interview process among primary health workers about how to make improvements for chronic illness care might have influenced health workers to put efforts in their day-to-day work to improve and this was subsequently reflected in their scoring in midline and end line.

The ACIC is also a useful tool to identify areas that need more focus for improvements [10]. The current study results clearly indicate that in the MHCP, more improvement is needed especially for the system related components of the CCM, such as organization of health care delivery system and delivery system redesign.

Our study also shows that the ACIC tool tailored for the management of mental illness in primary care can be used to assess the level of improvement in quality of care. However, to determine the extent of support for CCM elements in the community and to assess whether care did indeed improve as a result of the intervention, it would be important to administer the patient version of the ACIC tool–commonly referred to as the PACIC, among patients to determine whether the changes in the system have had an effect on their experience of care. This is important because patients and care givers may evaluate the chronic care elements differently to service providers [24]. This study was part of a larger initiative to evaluate the effectiveness of integrating mental health into primary health care. The main evaluation of MHCP is reported elsewhere [25] which also includes patients related outcomes.

The findings show primary health care workers’ positive assessment on improvements in chronic care elements as a result of the MHCP. The positive perception and motivation of primary health care workers is a precondition for successful mental health integration in chronic care. The findings derived from ACIC instruments provide useful implementation realities that other LMICs need to consider while implementing MHCP. These findings, however, should be interpreted in light of substantial financial, technical and coordination inputs provided by the implementing non-governmental organization, something that might not be feasible for regular government programs. The incentives to health service providers influence their behavior in chronic care [26] so that the sustainability of the achievements gained through the MHCP, would depend on the incentive mechanisms to motivate primary health workers.

Likewise, the results should be interpreted in light of power dynamics among several stakeholders involved in this study. In this project, all clinical work was done by government employed primary health workers with training and supervision support provided by Nepalese psychiatrists and clinical psychologists. This study was a collaboration between researchers from the global North (Europe) and the global South (Nepal and other 5 low and middle income countries in Asia and Africa) which ensured an open exchange between the researchers throughout the project.

### Strengths and limitations

A strength of this study was that the ACIC was administered in a group setting allowing for discussion and clarification. The ACIC assessment itself became an intervention as the discussion among the team members helped identify differing views of health workers and explore areas for improvement.

One of the limitations of the study is that due to the transfer of health staffs between baseline, midline and end line, not all the health workers who participated at baseline could take part at midline or end line. Some new health workers who joined the health facilities at midline and end line were included to develop a group response to the ACIC questions. Consequently, the group response might have been affected by the views of the new health workers. Another limitation of the study is that the threshold mentioned in the original English version of ACIC might not have reflected the actual threshold limit for Nepali population as the ACIC instrument was not validated for use in Nepal.

The possibility of confounders is another limitation of the study as the allocation of health facilities to the intervention and comparative control group was not randomized. All the intervention health facilities were situated in Western Chitwan, and comparative control group health facilities were in Eastern Chitwan, so the comparative control group might be systematically different from the intervention group. Another limitation is the possibility of respondent bias as respondents from the intervention group might be more inclined to give socially desirable responses. In future studies the findings from these ACIC scores could be triangulated with the findings from more in-depth anthropological work on perceived quality of services. The anthropological work would be helpful to explore how mental health is perceived in a collective society like Nepal and to what extend the mhGAP based mental health interventions are in compliance or in conflict with locally held beliefs, illness categories and modes of treatment.

Another limitation is that the analysis was not controlled for the site characteristics. Likewise, we did not perform a power analysis (eg. sample size calculation) because at the start of the study we lacked essential information to do a reliable power analysis. As this was the first study of its nature in Nepal, we had no indication of the ‘effect size’ of the ‘study intervention’ nor did we have the reference articles to base the sample size calculation and power analysis for this study. Finally, the study has limitations in terms of generalizability of the findings as the data were from only one geographic area (Chitwan district), with a relatively small number of health workers interviewed per health facility. However, this study has novelty as it is the first evaluation of collaborative chronic care for mental illness in Nepal, and its preliminary findings will guide future studies evaluating the process of integrating mental health into primary health care in a low income country setting.

Future research should include a larger number of health facilities with the geographical representation. The patients’ and caregivers’ perspectives on the perceived quality of services could be explored further. A separate study to validate the ACIC instrument in Nepal would be useful to reflect the threshold limit of ACIC for the Nepalese population.

## Conclusions

The primary health care workers reported improvements in perceived quality of care systems for mental illness in health facilities that received support under MHCP, compared to those that did not. At baseline there were no statistically significant differences between the intervention and comparative control groups but at midline and end line there was a significant difference in all six elements of the ACIC. Although the health facilities in both groups had improvements from baseline to end line, the health facilities in the intervention group significantly outperformed the comparative control group. This shows that the MHCP (including the training and supervision of health workers) contributed in strengthening the integration of chronic care elements in primary health care.

## Data Availability

The data is reported within this manuscript. Additional graphs/figures are provided in supplementary file. The dataset used in this study are available upon request from the corresponding author.

## Abbreviations

ACIC: Assessment of Chronic Illness Care
CCM: Collaborative Chronic Care
FCHVs: Female Community Health Volunteers
LMICs: Low and Middle Income Countries
MHCP: Mental Health Care Package
MhGAP: Mental Health Gap Action Program
MNS: Mental, Neurological and Substance Abuse
WHO: World Health Organization
